# Respiratory motion prediction based on deep artificial neural networks in CyberKnife system: A comparative study

**DOI:** 10.1002/acm2.13854

**Published:** 2022-12-01

**Authors:** Payam Samadi Miandoab, Shahyar Saramad, Saeed Setayeshi

**Affiliations:** ^1^ Department of Energy Engineering and Physics, Medical Radiation Engineering Group Amirkabir University of Technology Tehran Iran

**Keywords:** deep artificial neural network, hyperparameter, motion prediction, optimization, radiotherapy

## Abstract

**Background:**

In external beam radiotherapy, a prediction model is required to compensate for the temporal system latency that affects the accuracy of radiation dose delivery. This study focused on a thorough comparison of seven deep artificial neural networks to propose an accurate and reliable prediction model.

**Methods:**

Seven deep predictor models are trained and tested with 800 breathing signals. In this regard, a nonsequential‐correlated hyperparameter optimization algorithm is developed to find the best configuration of parameters for all models. The root mean square error (RMSE), mean absolute error, normalized RMSE, and statistical *F*‐test are also used to evaluate network performance.

**Results:**

Overall, tuning the hyperparameters results in a 25%–30% improvement for all models compared to previous studies. The comparison between all models also shows that the gated recurrent unit (GRU) with RMSE = 0.108 ± 0.068 mm predicts respiratory signals with higher accuracy and better performance.

**Conclusion:**

Overall, tuning the hyperparameters in the GRU model demonstrates a better result than the hybrid predictor model used in the CyberKnife VSI system to compensate for the 115 ms system latency. Additionally, it is demonstrated that the tuned parameters have a significant impact on the prediction accuracy of each model.

## 1 INTRODUCTION

Respiratory motions during the patient's breath can significantly affect the precision of radiotherapy when hypofractionated high‐dose radiotherapy is delivered to dynamic tumors, such as those in the lung, breast, liver, and pancreas. In this context, several compensation techniques, including breath‐holding methods, respiratory gating, and real‐time tumor‐tracking methods, have been clinically used for motion management.^1^, ^2^ Overall, the goal of respiratory gating or real‐time tumor tracking methods is to detect, predict, and compensate for respiratory motion during the planning and delivery of radiotherapy. However, the inherent delay present in modern radiotherapy equipment caused by data processing, saving, reading, radiation field adjustment, and communication with the robot controller affects the accuracy of radiation dose delivery.^3^, ^4^ In this regard, a compensation strategy is required in modern radiotherapy devices to minimize the effects of system latency. Currently, the latency in the advanced radiation therapy systems is about 50–500 ms^5^, ^6^; to correct this effect, a prediction model has been used in precise and advanced radiotherapy systems, such as the CyberKnife, Radixact, and Vero systems.^7^, ^8^

Between 1995 and 2012, three algorithms for predicting respiratory motion during treatment were proposed: model‐based, model‐free, and hybrid algorithms.^9^, ^10^, ^11^, ^12^, ^13^, ^14^, ^15^, ^16^, ^17^ The model‐based algorithms estimate the predictions of respiratory signals using a mathematical equation, whereas model‐free algorithms employ a heuristic learning algorithm to predict respiratory motion. In contrast, hybrid algorithms use integrated methods to combine model‐based and model‐free algorithms to predict the respiratory signals. In this context, a review of previous studies reveals the challenges faced by researchers, such as low accuracy and poor performance of models for irregular respiratory patterns, as well as ignorance of the respiratory signal's time dependence.^12^, ^13^, ^14^, ^18^, ^19^, ^20^, ^21^ To tackle the issue of ignorance of temporal dependence, a deep recurrent neural network (RNN) was proposed.^22^ However, deep RNN networks suffer from the problem of exploding and vanishing gradients, which makes it challenging to train long‐term sequences. To estimate the respiratory signal, a variety of models from the category of deep RNN networks, including long short‐term memory (LSTM), gated recurrent unit (GRU), bidirectional LSTM (Bi‐LSTM), and bidirectional GRU (Bi‐GRU) networks, have recently been proposed.^22^, ^23^, ^24^ The results of the cited studies show that the new approaches can predict respiratory signals with acceptable accuracy in comparison to the previous algorithms. For example, Lin et al. presented a robust static neural network (LSTM model), which was accompanied by sequential hyperparameter optimization (HPO) to predict respiratory motion with short system latency.^22^ The obtained results are much better than the algorithms mentioned earlier; however, there are still some significant limitations that require further investigation. The limitations of cited studies include considering few parameters for optimization, the lack of comprehensive comparisons between other deep learning networks, and the lack of assessment of the impact of the correlated hyperparameters on network accuracy. In this work, three key challenges are believed to require more attention: (1) studying the patient's respiration as it varies significantly from patient to patient, (2) investigating the interaction of parameters in the network using optimization algorithms to improve the accuracy and reduce the training time of the model, and (3) proposing a robust network that can predict short‐ and long‐term changes in respiratory motion during treatment.

In this study, seven deep learning methods, including simple RNN, LSTM, GRU, Bi‐simple RNN, Bi‐LSTM, Bi‐GRU, and CNN‐LSTM, are considered to predict the respiratory signals. Overall, the experiment was conducted in two steps. In the first step, an attempt was made to select the best configuration of parameters for all networks using a nonsequential‐correlated HPO algorithm. Specifically, the parameters considered are the number of layers, number of hidden units in each layer, learning rate, type of activation function in hidden and output layers, number of epochs, system latency, optimizer, batch size, loss function, input‐slide windowing, output‐slide windowing, and multistep types. Subsequently, the second step involved a comprehensive comparison of the seven deep learning methods to find the best predictive model. The employed deep learning networks were trained with CyberKnife VSI Synchrony motion data from 30 patients. The root mean square error (RMSE), mean absolute error (MAE), normalized root mean square error (NRMSE), and statistical *F*‐test are also considered to evaluate the performance of the seven deep learning networks.

## 2 MATERIALS AND METHODS

### 2.1 Data acquisition and preprocessing

**FIGURE 1:**
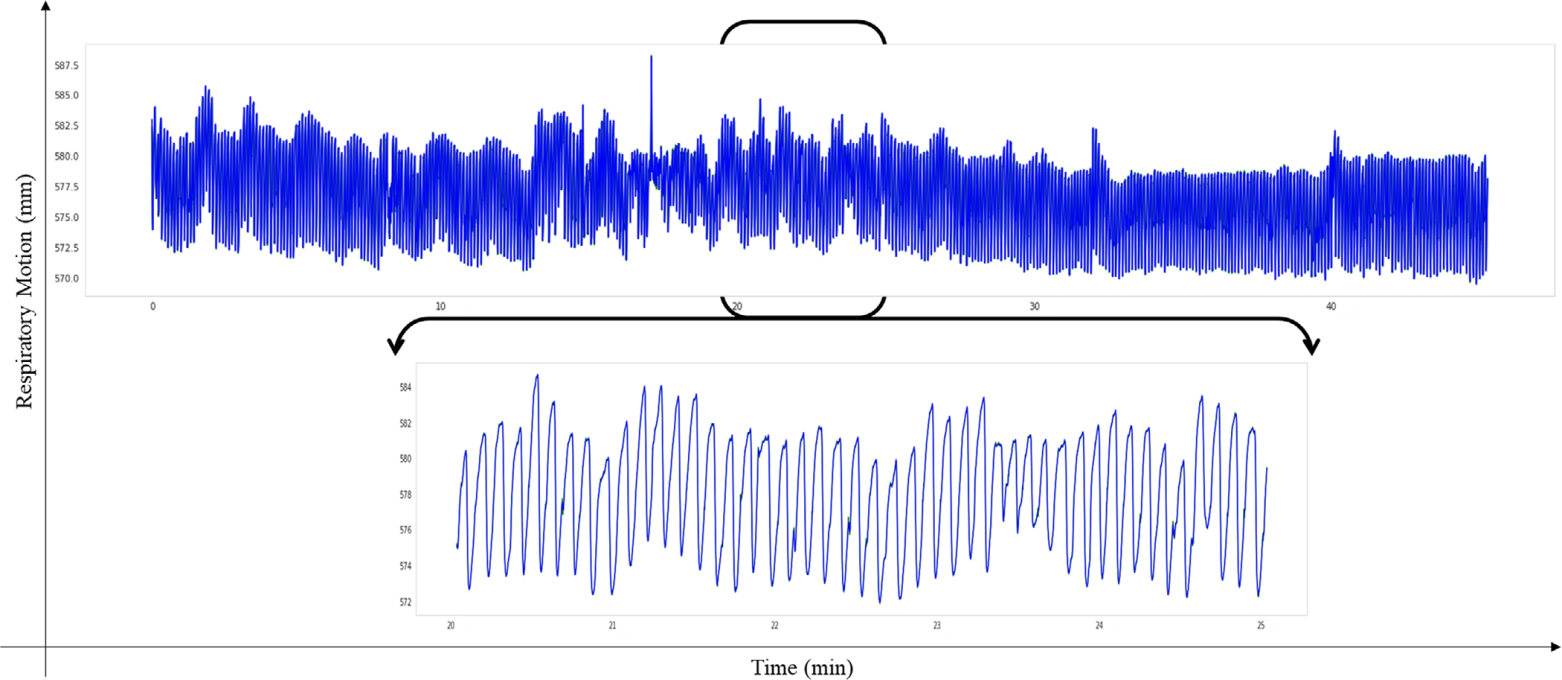
The breathing signals (mm) obtained from the external marker

Respiratory motion data were collected from 30 lung and abdominal cancer patients treated with the CyberKnife VSI system—version 9.5 (Accuray, Inc., Sunnyvale, CA, USA). This database contains 97 treatment fractions of respiratory motion data with the frequency of 26 Hz decomposes into steady breathing, slow and increased depth of breathing, slow and shallow depth of breathing, rapid and increased depth of breathing, and rapid and shallow depth of breathing. The treatment characteristics, including the tumor sites, number of patients, number of treatment fractions, average dose (Gy), and target tracking system, are shown in Table 1. The system latency of the CyberKnife VSI system is also 115 ms, and the recorded length of the respiratory ranged from 23 to 60 min, with a sample of the breathing signals (mm) obtained by the external marker shown in Figure 1. Note that, in the preprocessing step, the data normalization transforms the data into a specific range: 0–1. Performing the data normalization step reduces the number of computations of the deep learning network, improves the convergence speed, and increases the algorithm's accuracy.

**TABLE 1:** Features of the case studies, including tumor sites, number of patients, number of treatment fractions, average dose (Gy), and target tracking system for 30 patients (97 treatment fractions) treated by the CyberKnife VSI system

Tumor location	Number of patients	Treatment fractions	Average dose (min–max) (Gy)	Systems
Lower left lung	2	7	38.13 (19–49)	XLT
Lower right lung	2	6	38.63 (20–54)	XLT
Upper left lung	3	10	41.4 (20–60)	XLT
Upper right lung	7	23	38.68 (20–51)	XLT
Central liver	5	18	39.11 (24–60)	FTT
Lower liver	4	10	40.37 (30–45)	FTT
Upper liver	7	23	42.51 (30–45)	FTT

*Note*: Fiducial‐based target tracking (FTT) and Xsight Lung Tracking (XLT) systems.

### 2.2 Structure of Synchrony Respiratory Tracking System (SRTS) in the CyberKnife system

**FIGURE 2:**
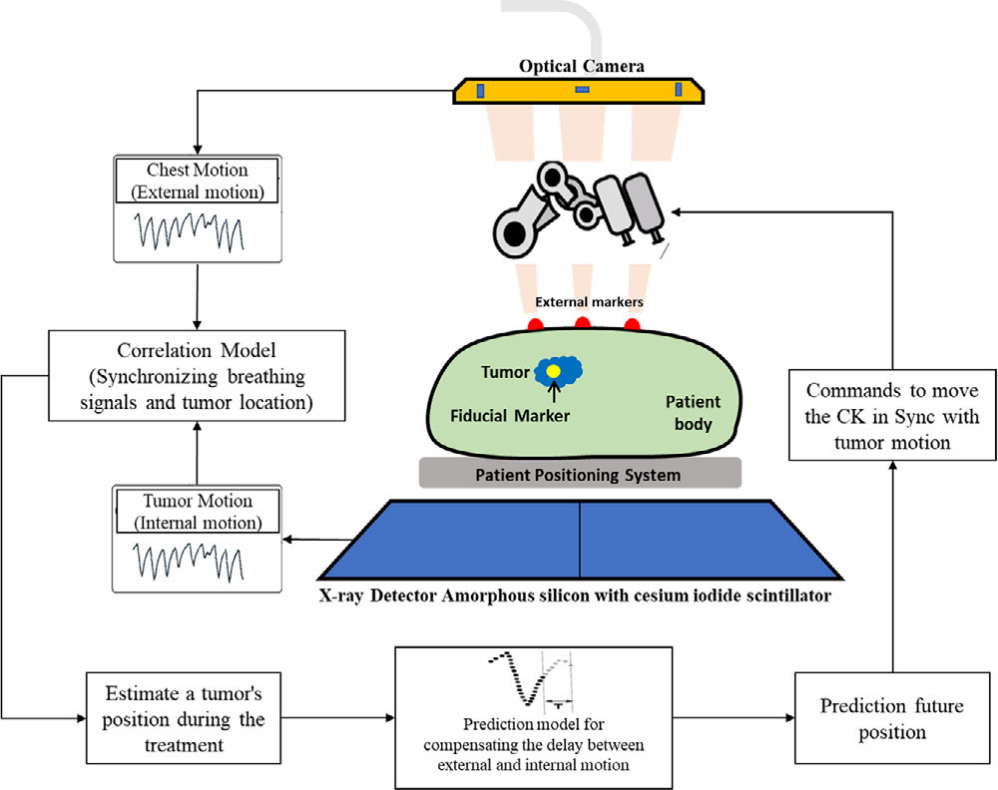
Schematic representation of the Synchrony Respiratory Tracking System (SRTS) in the CyberKnife system

The Synchrony Respiratory Tracking System (SRTS) in the CyberKnife system uses correlation and prediction models to perform real‐time tumor tracking (Figure 2). Therefore, the SRTS technology provides an ability to steer the external beams continually to the tumor with motion caused by breathing throughout the treatment. The fundamental concept of the SRTS is synchronizing the respiratory with the tumor position. Although the breathing signal is monitored every 10–40 ms through the optical camera system, the target and marker positions are determined per X‐ray image acquisition.^3^, ^8^ Therefore, a correlation model can track the tumor movement using the correlation between the patient's respiration and tumor position. The correlation model is also updated every 1–6 min throughout treatment using periodic X‐ray images. Note that the 115 ms system latency, due to robotic response or data acquisition, affects the accuracy of radiation dose delivery.^3^ Therefore, the Synchrony system employs a predictive model to correct this effect. In this study, seven prediction models, including simple RNN, LSTM, GRU, Bi‐simple RNN, Bi‐LSTM, Bi‐GRU, and CNN‐LSTM, are considered to compensate for the manipulator time lag. In Supporting Information, these models are shortly discussed.

The data partitioning of the respiratory breathing signal, including training, validation, and testing, is divided into 35%, 5%, and 60% of the individual respiratory. Furthermore, all of the models include a time‐domain input‐sliding window. The input‐sliding window provides additional information for the network structure to improve the prediction accuracy during training. Note that all prediction models were trained without updating the model weights during the respiratory signal prediction. Section 2.3 also describes the HPO for all networks used.

### 2.3 Hyperparameter optimization (HPO)

**FIGURE 3:**
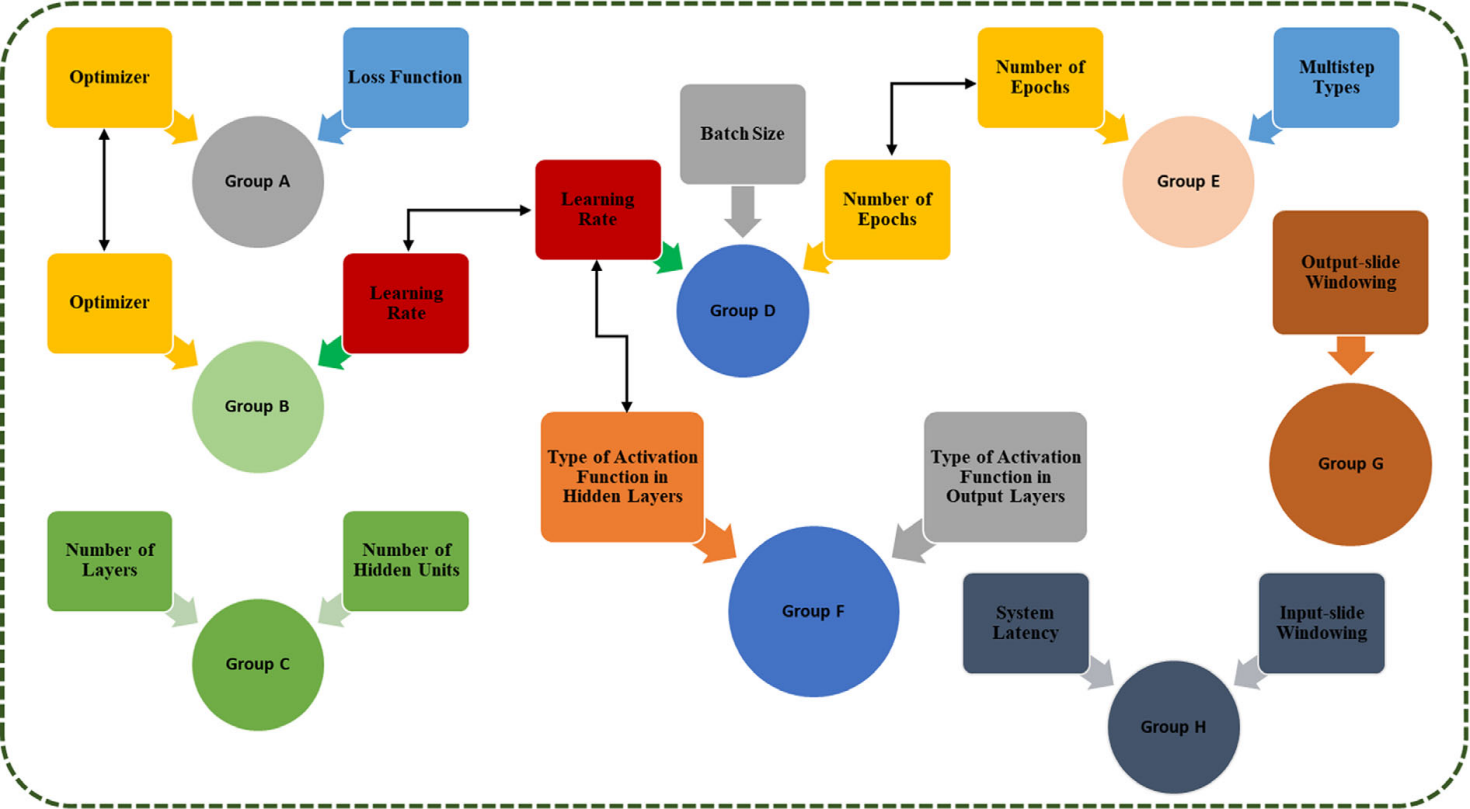
The flowchart of the nonsequential‐correlated hyperparameter optimization algorithm. The double black line arrow depicts the type of indirect link between different parameters.

HPO is a process for selecting a set of optimal hyperparameters to achieve maximum accuracy with the network. Moreover, the selection of the best hyperparameters affects both the training speed and the network structure. In this regard, search algorithms and trial schedulers are two state‐of‐the‐art HPO methods.^25^ Although the search algorithm is an exhaustive search through the user‐specified parameters, the trial method is primarily concerned with the method of early stopping of model evaluation. In this study, a nonsequential‐correlated HPO algorithm was developed to find the best configuration for all models. Figure 3 depicts the proposed algorithm's workflow. Based on this figure, for different groups, small grid or random search algorithms are defined, which are used for nonsequential or nonsequential‐correlated optimizations, respectively. In Group C, for example, the best configuration between the number of layers and the number of hidden units is determined using a small grid search algorithm (Figure 3). It should be noted that some parameters, such as the optimizer, learning rate, and the number of epochs, have an indirect relationship with others, which are depicted by the double black line arrow in Figure 3. Therefore, optimizing these parameters is necessary. For example, after determining a trade‐off between the loss function and the optimizer in Group A, the relationship between the type of optimizer and the learning rate is investigated in Group B, which is based on the small random search algorithm (Figure 3). By performing this process, the algorithm focuses the search on the most promising parameters. In total, 30 000 random searches were performed in this study for 12 variants, each with 10 trials. Supporting Information contains additional information on these parameters.

## 3 EVALUATING THE ACCURACY OF PREDICTION MODELS

Four performance metrics, including RMSE, MAE, NRMSE, and statistical *F*‐test, are considered to evaluate the performance of the predicted respiratory signal. In this series, the RMSE is considered a measurement of the differences between the predicted and the actual values, which is defined as a mathematical equation by

(1) $$RMSE\;=\;\sqrt{\frac{1}{N}\;\sum_{i\;=\;1}^{N}{\left(Y_{i}-\;\hat{Y_{i}}\right)}^{2}}$$

The MAE, on the other hand, is used to precisely reflect the actual prediction error between two continuous variables in time‐series analysis. The MAE, the average of the absolute values, represents the deviations between paired observations expressing the same phenomenon. The mathematical expression for the MAE is

(2) $$MAE\;=\;\frac{1}{N}\;\sum_{i\;=\;1}^{N}\left|Y_{i}-\;\hat{Y_{i}}\right|$$

Because our data are time‐series datasets, the RMSE is not reliable or appropriate for evaluating the performance of the numerical model. In this relation, the NRMSE is proposed as a suitable approach to compare the RMSE of the prediction model with different scales of breathing signal. In other words, because normalizing the RMSE makes the RMSE scale‐free, the NRMSE is a better indicator for evaluating the model performance. By converting NRMSE to a percentage, the model's performance becomes comparable to the variability of respiratory signals with different amplitudes. In this context, the lower NRMSE shows lower variance. The NRMSE is calculated by

(3) $$NRMSE\;=\frac{RMSE}{Y_{\;max}\;-\;Y_{\;min}}$$

Statistical *F*‐test analyses were also performed for all models to test a significant statistical association between the RMSE of the real value ($Y_{i}$) and the RMSE of the predicted value ($\hat{Y_{i}}$). A *p*‐value less than 0.05 was also considered statistically significant.

Among the above expressions, *N* is the number of investigated points, $Y_{i}$ is the ground‐truth values, $\hat{Y_{i}}$ is the predicted values of the model, $Y_{max}$ is the maximum value of the ground‐truth values, and $Y_{min}$ is the minimum value of the ground‐truth values.

## 4 EXPERIMENT PLATFORM

In all structures, the trained and tested models were implemented in the Python environment (version 3.7) using the high‐level neural network API Keras (version 2.4) and the back end engine TensorFlow (version 2.4).

## 5 RESULTS

In the present work, a nonsequential‐correlated HPO algorithm was developed to find the best configurations for all models. This algorithm investigated the interaction of correlated parameters that have a significant impact on the model's performance. Table 2 summarizes the grid search parameters for all models, the final selected configuration, and their importance levels. Overall, the tuned parameters can be classified into five categories: very high, high, medium, low, and very low impacts. The high impacts, including the number of hidden units per layer, the epoch number, and the optimizer, play an important role in model performance and accuracy. On the other hand, a number of layers and batch size have little impact on a given network's performance. The type of activation function in hidden and output layers, learning rate, input‐ and output‐sliding window, and multistep types have a medium effect on the obtained results. In this series, the system latency with a significant impact on the network performance and loss function with a minor impact are categorized in the very high and very low impacts, respectively.

**TABLE 2:** The lists, ranges of values, selected configuration, and impact level of each parameter used in the grid search for all models

		Simple RNN		LSTM		GRU		Bi‐simple RNN		Bi‐LSTM		Bi‐GRU		CNN‐LSTM	
Parameter	Range for all networks	Recommended configuration	Impact	Recommended configuration	Impact	Recommended configuration	Impact	Recommended configuration	Impact	Recommended configuration	Impact	Recommended configuration	Impact	Recommended configuration	Impact
Number of layers	1, 2, 3, 5	2	Low	2	Low	3	Low	3	Low	2	Low	3	Low	1	Low
Number of hidden units per layer	3, 5, 10, 20, 30, 40, 50, 60	10	High	10	High	10	High	5	High	5	High	5	High	20	High
Optimizer	SGD, Adam, Adamax, Nesterov Adam, Adagrad, Adadelta, RMSprop, FTRL	Adam	High	Adam	High	Adam	High	Adam	High	Adam	High	Adam	High	Adam	High
Learning rate	0.0001, 0.005, 0.001, 0.005, 0.003, 0.05, 0.01	0.001	Medium	0.005	Medium	0.005	Medium	0.001	Medium	0.005	Medium	0.003	Medium	0.003	Medium
Activation function in hidden layers	Swish, Sigmoid, Relu, Selu, Elu, Softsign, Tanh, Softmax, Softplus, Hard Sigmoid, Linear	Swish	Medium	Relu	Medium	Elu	Medium	Tanh	Medium	Softsign	Medium	Softsign	Medium	Tanh	Medium
Activation function in output layer	Linear, Sigmoid, Swish, Softsign	Linear	Medium	Linear	Medium	Linear	Medium	Softsign	Medium	Linear	Medium	Sigmoid	Medium	Swish	Medium
Number of epochs	125, 250, 500, 1000, 2000	500	High	500	High	1000	High	500	High	500	High	1000	High	500	High
System latency	1,5, 10, 15	1	Very high	1	Very high	1	Very high	1	Very high	1	Very high	1	Very high	1	Very high
Batch size	50, 100, 150, 200, 250, 300, 400, 500	300	Low	250	Low	200	Low	300	Low	200	Low	200	Low	200	Low
Loss function	MAE, MSE, Huber, LogCosh	MSE	Very low	MSE	Very low	MSE	Very low	MAE	Very low	MAE	Very low	MAE	Very low	MAE	Very low
Input‐slide windowing	1, 5, 10, 20, 30, 50, 100	50	Medium	50	Medium	50	Medium	50	Medium	50	Medium	50	Medium	50	Medium
Output‐slide windowing	1, 3, 5, 10	1	Medium	1	Medium	1	Medium	1	Medium	1	Medium	1	Medium	1	Medium
Multistep types	Encoder–decoder, vector	Encoder–decoder	Medium	Encoder–decoder	Medium	Encoder–decoder	Medium	Encoder–decoder	Medium	Encoder–decoder	Medium	Encoder–decoder	Medium	Encoder–decoder	Medium

Abbreviations: Bi‐GRU, bidirectional GRU; Bi‐LSTM, bidirectional LSTM; GRU, gated recurrent unit; LSTM, long short‐term memory; MAE, mean absolute error; RNN, recurrent neural network.

**FIGURE 4:**
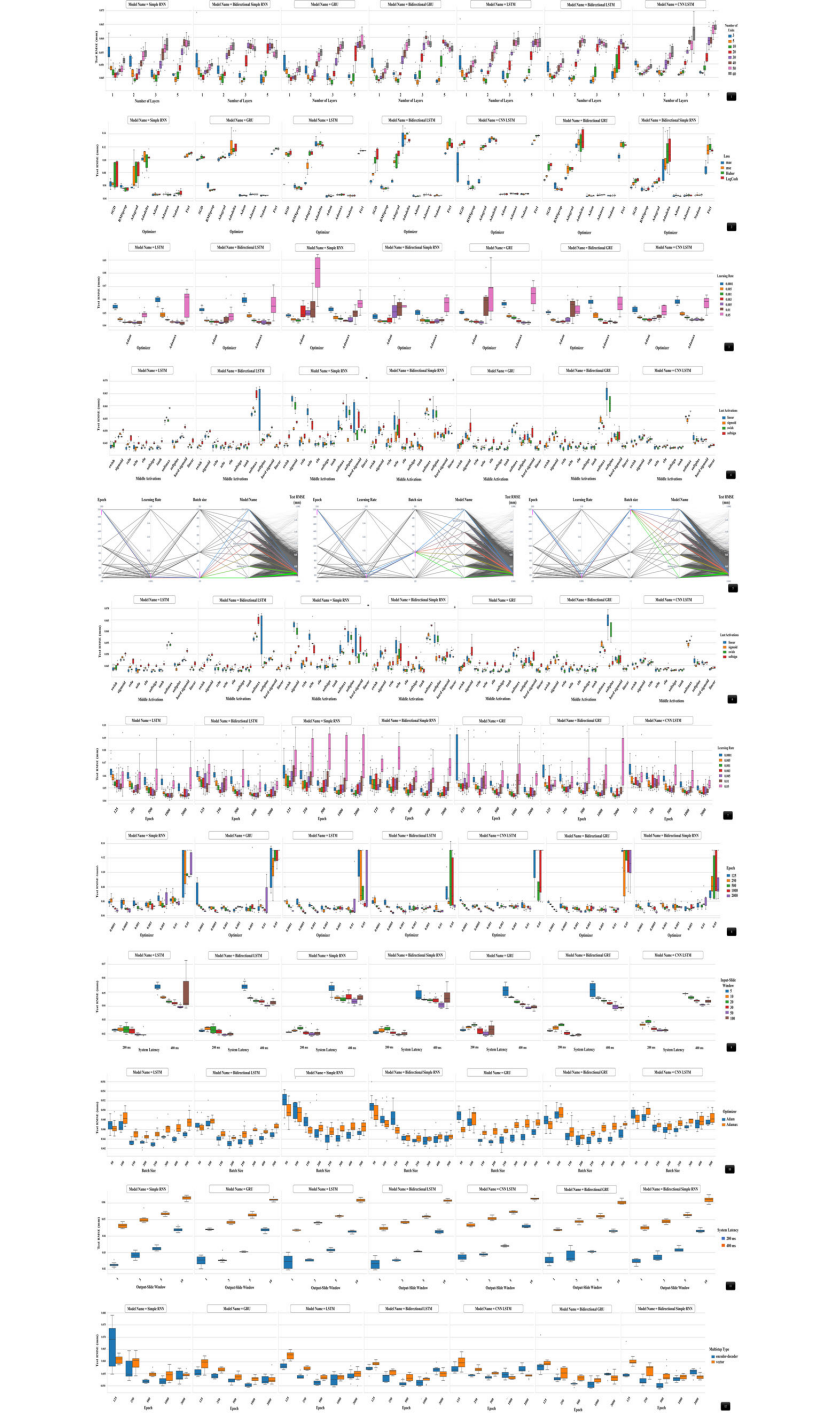
Interaction levels of different hyperparameters on the accuracy of the predictive models (note that because the word inherently reduces the image quality, please download the original image file)

The obtained results from the optimization algorithm were also used to investigate the interaction levels of some hyperparameters for each of the seven models. It is believed that, by using the proposed approach, better results can be obtained in comparison to the previous studies, which used individual parameter optimization. In Figure 4, the RMSE is plotted as a function of the number of layers, number of hidden units in each layer, learning rate, type of activation function in hidden and output layers, number of epochs, system latency, optimizer, batch size, loss function, input slide windowing, output slide windowing, and multistep types to evaluate the performance of all the models. In order to provide better interpretation of the impact of the parameter interaction levels on the accuracy and performance of the different models, a comparison between some hyperparameters was also considered.

**FIGURE 5:**
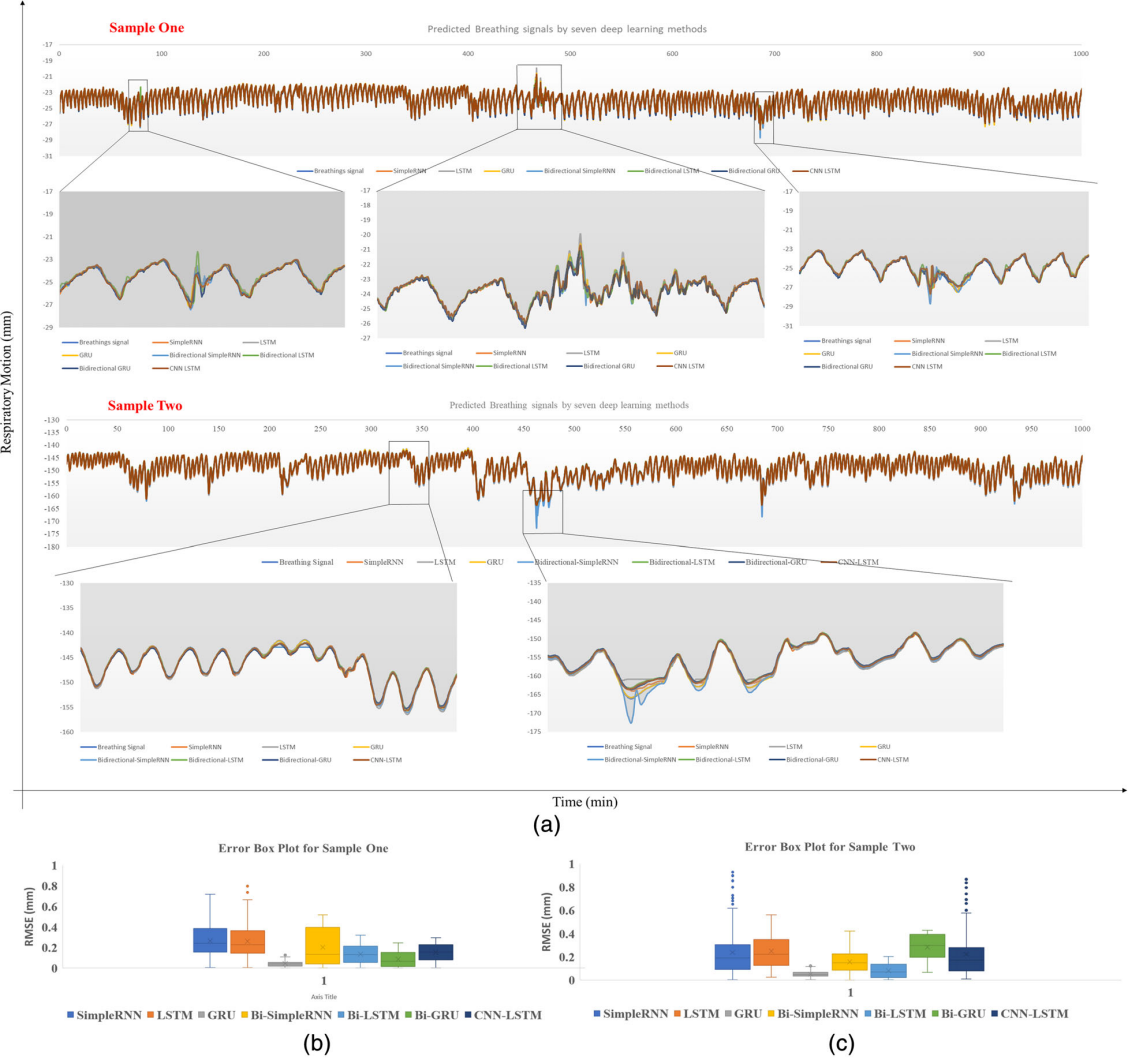
(a) The comparison of predicted respiratory signals (two different samples) by all models: (1) simple recurrent neural network (RNN), (2) long short‐term memory (LSTM), (3) gated recurrent unit (GRU), (4) Bi‐simple RNN, (5) bidirectional LSTM (Bi‐LSTM), (6) bidirectional GRU (Bi‐GRU), and (7) CNN‐LSTM (note that because the word inherently reduces the image quality, please download the original image file); (b) error box plot (root mean square error [RMSE] value) for predicted sampled one; (c) error box plot (RMSE value) for predicted sampled two. (Note that the box shows the median, 25th, and 75th percentiles. The dotted whisker line extends to the most extreme data point (field), not considered outliers. Outliers are defined as points positioned more than 2.7 standard deviations from the mean.)

Following the determination of the best configuration for all networks, 800 breathing signals from 30 patients were examined to determine the accuracy and performance of the prediction models. Due to the high variability of the respiratory signals, the NRMSE was also used in this study to develop a better understanding about the variability in the respiratory signals. In this context, the lower NRMSE shows more reliable prediction. Based on Table 3, all the models can be used in practice; however, the GRU model, based on train and test results, can provide a better result and performance. In addition, for all models, the hypothesis of a significant statistical relationship between the RMSE of the real value and the RMSE of predicted value is tested. For this purpose, the *F*‐test with a *p*‐value test less than 0.05 is considered to check the statistical significance of the relationship. Overall, all models pass the *F*‐test with good statistics. Additionally, for a given patient, by using all models, a comparison of predicted respiratory signals and error box plot (RMSE Value) with two different samples (between 25 and 40 min) is shown in Figure 5.

**TABLE 3:** Comparing the performance of different prediction models; the mean ± standard deviation (SD) of the root mean square error (RMSE) (mm), mean absolute error (MAE) (mm), and normalized root mean square error (NRMSE)

		Simple RNN	LSTM	GRU	Bi‐simple RNN	Bi‐LSTM	Bi‐GRU	CNN‐LSTM
Train	RMSE (mm)	0.109 ± 0.065^a^	0.119 ± 0.08^a^	0.085 ± 0.043^a^	0.125 ± 0.079^a^	0.121 ± 0.067^a^	0.135 ± 0.099^a^	0.128 ± 0.076^a^
	MAE (mm)	0.079 ± 0.049	0.083 ± 0.052	0.066 ± 0.037	0.095 ± 0.07	0.089 ± 0.045	0.11 ± 0.063	0.111 ± 0.066
	NRMSE	0.027	0.032	0.023	0.033	0.032	0.035	0.035
Test	RMSE (mm)	0.131 ± 0.083^a^	0.146 ± 0.089^a^	0.108 ± 0.068^a^	0.155 ± 0.096^a^	0.149 ± 0.09^a^	0.185 ± 0.088^a^	0.189 ± 0.099^a^
	MAE (mm)	0.099 ± 0.059	0.102 ± 0.065	0.086 ± 0.045	0.119 ± 0.066	0.105 ± 0.08	0.131 ± 0.078	0.144 ± 0.067
	NRMSE	0.038	0.039	0.031	0.04	0.039	0.047	0.049

Abbreviations: Bi‐GRU, bidirectional GRU; Bi‐LSTM, bidirectional LSTM; GRU, gated recurrent unit; LSTM, long short‐term memory; RNN, recurrent neural network.

^a^ Statistical *F*‐test analyses by considering a *p*‐value less than 0.05 were performed for all models to test a significant statistical association between the RMSE of the real value ($Y_{i}$) and the RMSE of the predicted value ($\hat{Y_{i}}$).

## 6 DISCUSSION

### 6.1 Hyperparameter optimization (HPO)

Examining variables that appear frequently or rarely in the same graph can help in identifying the potential gaps in the network structure, which is necessary when developing a deep learning model. In this context, a review of previous studies shows the use of an individual hyperparameter tuning approach for network structure.^22^, ^23^, ^24^ In practice, however, some correlated hyperparameters can have a significant impact on network performance. Therefore, the HPO algorithm is imperative for determining the optimal network configurations in order to improve prediction accuracy and training speed. In this study, 12 variants were considered; however, considering the combination of all variables results in 700 million combinations. Because considering 700 million combinations are time‐consuming and beyond the scope of this work, a nonsequential‐correlated HPO algorithm is developed to find the potentially interesting combinations, which results in 30 000 iterations. This algorithm is based on the relationship between correlated irrelevant parameters. The results show that hyperparameter tuning improved the performance of all models by 25%–30% when compared to previous studies. The following are the proposed hyperparameters used in this study:

1. The number of layers: As the number of layers required in a network is determined by several factors, including the number of features, the complexity of the dataset, the number of samples, and other network hyperparameters, there is no set rule for determining the number of layers in the network. Generally, different layers and configurations are expected to be proposed for each model; nevertheless, most problems can be handled with two to three layers of the network. Overall, tuning the number of layers improves the results by roughly 15%.
2. The number of hidden units in each layer: The model width is determined by the number of hidden units per layer. According to the results shown in Figure 4‐1, there is a relationship between the number of layers and the number of hidden units in each layer. Overall, the number of hidden units per layer affects the model performance more than the number of layers. In this study, for a network with two to three layers, it is reliable to use a few hidden units per layer. Overall, a value of 10 or 20 appears to be a good starting point for network hidden units.
3. Optimizers: Another hyperparameter related to improving accuracy and training speed is the optimizers or optimization algorithms. The optimizer has a direct impact on the network performance. This study takes into account a variety of optimizers, including SGD, Adam, Adamax, Nesterov Adam, Adagrad, Adadelta, RMSprop, and FTRL. Based on the currently available data, it appears that Adamax and Adam performed the best overall, whereas SGD, Adagrad, Adadelta, and FTRL took a longer time to converge (Figure 4‐2). The detailed analysis also demonstrates that Adamax and Adam, with a learning rate ranging from 0.001 to 0.005, accelerate the convergence and have better performance (see Figure 4‐3). In this study, the interaction levels of the two recommended optimizers with different types of activation functions in the hidden layer were investigated. According to Figure 4‐4, the influence of the two recommended optimizers on the middle activation function has a minor impact on the network performance. Note that using other optimizers may result in different performances and needs more consideration to avoid the possibility of vanishing gradient problems.
4. The learning rate: In reality, determining the best learning rate is a difficult and time‐consuming task. Based on Figure 4‐5, although the learning rate has a strong impact on the network accuracy when the epoch number is less than 500, increasing the epoch number has a moderate impact. Moreover, when using a higher or lower epoch, the batch size has a limited impact on the learning rate (Figure 4‐5); otherwise, the batch size has a moderate impact on the network performance. In this figure, three batch sizes are considered: 100, 256, and 500. Interestingly, the learning rate is also affected by the type of optimizer used (Figure 4‐3). In this study, the impact of the two recommended optimizers on the performance of various learning rates, including 0.0001, 0.0005, 0.001, 0.003, 0.005, 0.01, and 0.05, was also investigated. The results demonstrated that the optimal values for the learning rate are 0.001 and 0.005. In this series, the optimizer's interaction with the learning rate has a medium impact on the results.
5. The type of activation function in the hidden and output layers: The activation function is a mathematical operation that provides complex mappings between inputs and outputs, which is essential, sensitive, and crucial for modeling and learning complex data. In addition, different activation function configurations in the hidden and output layers can result in different performances. In this study, Swish, Sigmoid, Relu, Selu, Elu, Softsign, Tanh, Softmax, Softplus, Hard Sigmoid, and Linear were studied in the hidden layers, and Linear, Sigmoid, Swish, and Softsign were studied in the output layers. According to Figure 4‐6, the Swish, Relu, Elu, Softsign, and Tanh activation functions perform better in the hidden layers, whereas the Linear or Swish activation functions perform better in the output layers. Overall, all of the output layer activation functions are practical.
6. The number of epochs: Because the number of epochs is a hyperparameter that affects the computed accuracy and model performance, it must be considered. Figure 4‐7 shows that using smaller or larger epoch numbers beyond a certain threshold (approximately 250 or 750 epochs, respectively) affects the prediction model's accuracy. Overall, an epoch number of 500 can provide reliable results for various networks. Moreover, as shown in Figure 4‐8, the epoch number corresponds to the learning rate. Therefore, it is critical to select an appropriate epoch number based on the optimal learning rate to avoid under‐ or overfitting. In the item 4 of the list, we indicate that the batch size also has a minor impact on the network performance when the epoch number is too high or too low; otherwise, the batch size had a medium impact on the network performance.
7. The system latency: System latency has a much larger impact on the accuracy and performance of the model than any other parameter. In this study, the effect of the system latency is comprehensively investigated by considering different values. The obtained results in Figure 4‐9 show that a larger system latency degraded the predictive model's accuracy and decreased the probability of convergence. Therefore, lower system latency is required in practice.
8. The batch size: The batch size allows for the updating of the network's internal parameters via training datasets. In this study, the effects of different batch sizes, including 50, 100, 150, 200, 250, 300, 400, and 500, were investigated. The results show that when the batch size is too small or too large, the accuracy of the predicted signals suffers. It must be noted that tasks with small training datasets require a smaller batch size, whereas tasks with large training datasets require a larger batch size. Moreover, lower batch sizes are more time‐consuming; therefore, a trade‐off between the optimal batch size and the model run‐time is required. Overall, it is found that the batch size is also affected by the optimizer types (see Figure 4‐10). In the item 3 of the list, we mentioned that Adamax and Adam provided the best performance; however, when batch size is taken into account, the results show that Adam is capable of providing better results than Adamax.
9. The Loss function: Studying the impact of the loss function resulted in only a minor improvement in network performance. As shown in Figure 4‐2, four types of loss functions are proposed: MAE, MSE, Huber, and LogCosh. Generally, MAE and MSE are two common types of loss functions. This study also looks into the relationship between the loss function and the optimizer. An examination of currently available data demonstrates that there is no need to find a trade‐off between optimizer and loss function because loss function has no significant impact on the performance of the optimizer.
10. The input‐slide windowing: As our data comprise a sequence of numbers ordered by a time index, the time‐series data must be converted into supervised data, which is made up of pairs of input data sequences, before being applied to the network. Using an input‐sliding window provides more information to the training network, which may improve prediction accuracy. However, a larger input‐sliding window increases the convergence time and may result in overfitting (see Figure 4‐9). There is also a link between the system latency and the input‐slide windowing. For higher system latency, a larger input window is required (see Figure 4‐9). As, in this task, the inherent system latency is 115 ms, an input‐slide windowing of 30 samples performs better.
11. The output‐slide windowing: An output‐sliding window refers to the number of time steps predicted by the model in each prediction step. As illustrated in Figure 4‐11, a larger output‐sliding window degrades the prediction model's accuracy. Therefore, in practice, a relatively small output‐slide windowing is required. In addition, this result contradicts previous reports by Mafi et al.^26^
12. Multistep types: For the network structure, two multistep types, vector and encoder–decoder models, are considered. Overall, the obtained results show that the multistep types had a moderate impact on the network's accuracy. Furthermore, when sequence‐to‐sequence prediction is required, the encoder–decoder model outperforms the network (see Figure 4‐12). A relation between multistep types and the number of epochs is also extracted. Overall, the encoder–decoder model converged quickly in a few epochs and outperformed the vector model. Using 500 epochs generally improves network performance; however, using a larger or smaller number of epochs may result in under‐ or overfitting, respectively.

### 6.2 Comparison of performance of the seven deep learning models

The aim of the second part of this study is to provide a comprehensive comparison between different models. In this context, seven deep learning models, including simple RNN, LSTM, GRU, Bi‐simple RNN, Bi‐LSTM, Bi‐GRU, and CNN‐LSTM, using the recommended configurations, were evaluated. The models were also tested using 800 different breathing signals. Because respiration is complex and nonstationary, the best prediction procedure must be able to work with the entire respiratory behavior spectrum. The recorded dataset in this study ranged from 23 to 60 min, and the recordings included all types of breathing patterns. Because previous studies only used a small number of patients’ breaths,^24^, ^27^, ^28^ consideration of such a robust dataset in this study is a great advantage.

In model analysis, four factors are considered: accuracy, precision, robustness, and generality. Accuracy refers to how close a result is to the true value, whereas precision is how close results are to each other. Robustness is defined as the model's ability to adapt to all types of breathing patterns without failing to predict the breathing pattern (a zoomed‐in portion of Figure 5a). Moreover, the error box plot (RMSE value) shown in Figure 5b,c shows the median, 25th, and 75th percentiles. The dotted whisker line extends to the most extreme data point (field), which is not considered an outlier. Outliers are also defined as points that deviate from the mean by more than 2.7 standard deviations. The ability of the model to handle a wide range of signal data without requiring special tuning for different types of signals to improve performance is referred to as generality. In this study, the results of the GRU model demonstrated more robust adaptability to observed breathing patterns (Figure 5). The GRU model (RMSE = 0.108 ± 0.068 mm), along with the recommended configuration, can make superior temporal predictions of breathing motion. In this relation, the reported results by Nakayama et al.,^29^ who analyzed the clinical log data of the predictor log file in the CyberKnife VSI system, show the radial prediction error to be 0.14 ± 0.11 mm. However, the results in this study reveal an almost 23% improvement in prediction accuracy compared to the hybrid model used in the CyberKnife system. Further details about hybrid models are also described in Sayeh et al.^30^ Interestingly, the RMSE result reported for the GRU model in this study is for a recorded dataset length ranging from 23 to 60 min, whereas previous studies used shorter recording lengths.^22^, ^24^ Yu et al., for example, trained the LSTM, GRU, and Bi‐GRU networks on six patients with recorded lengths ranging from 15 to 20 min, and they reported RMSE values of 0.14 ± 0.032, 0.15 ± 0.03, and 0.12 ± 0.034 mm, respectively.^24^ Lin et al. also used the LSTM network, which was tuned using a grid search, and they reported an RMSE value of 0.139 ± 0.085 mm for recorded lengths ranging from 2 to 5 min.^22^ In comparison to other studies, the proposed GRU model is more realistic. In this study, to provide a better concept due to the high variability of the respiratory signals, the NRMSE analysis is also considered. Our estimated NRMSE = 0.031 for the GRU model also shows a better result in comparison to the recent study by Jöhl et al., who investigated 18 prediction filters with time delays of 160 ms and reported an NRMSE value below 0.05 for both the wavelet least mean squares prediction filter and the linear filter.^31^

## 7 LIMITATIONS

There are a few limitations to this study that should be addressed in future research. The first limitation stems from the optimization algorithms of current method, which was developed to investigate the interaction levels of some hyperparameters in the seven models. As the combination of 12 variables results in 700 million combinations, a nonsequential‐correlated HPO algorithm is developed to find the potentially interesting combinations. Moreover, the implemented optimization algorithm lacks a thorough evaluation of other hyperparameter interactions that were not directly combined. Therefore, other optimization methods, such as Bayesian optimization, that can efficiently search the incorporated parameter interactions and the parameter space^32^ are needed to be investigated in future work. The second is about the results of the proposed optimization algorithm, which can be affected by the respiratory motion datasets. In this relation, a full length of breathing signals (50 min) that includes all types of variability of respiratory motions is considered. However, considering other datasets from different patients could result in different combinations and patterns, which will be investigated in the future study. The last limitation applies to all prediction models that were trained without updating the model weights during respiratory signal prediction. At real treatment using the CyberKnife system, to check the accuracy of model performance, an update will be performed during total treatment. In this relation, it is better that other parameters, such as updating time intervals and the number of datasets for each update, are considered, which will be investigated in the future study.

## 8 CONCLUSION

A nonsequential‐correlated HPO algorithm is developed to investigate and select the optimal configuration of the incorporated parameters in the seven deep learning artificial networks. Among all hyperparameters, the interaction of system latency, optimizers, number of hidden units per layer, and learning rate with input windowing, learning rate, number of layers, and number of epochs, respectively, required more consideration. To the best of our knowledge, this study is one of the first attempts to use a hyperparameter algorithm to investigate the impact of including parameter interactions on the prediction accuracy of the different models.

This paper presents several offline prediction models to compensate for system latency. Therefore, all prediction models were trained to predict respiratory signals once before treatment. In this context, 800 breathing datasets collected from 30 patients are also used to assess the performance of the seven deep learning models. Generally, it must be demonstrated that the tuned parameters have a significant impact on the prediction accuracy of each model. When all models are compared, the GRU model predicts respiratory signals with high accuracy, precision, robustness, and generality.

## AUTHOR CONTRIBUTIONS

All listed authors contributed to the study and to drafting the manuscript.

## CONFLICT OF INTEREST

The authors declare that they have no known conflict of interest or personal relationships that could have appeared to influence the work reported in this paper.

## ETHICS STATEMENT

The ethics committees of the University of Kiel and the University of Frankfurt have approved the use of anonymized data within the framework of the clinical evaluation of the CyberKnife Synchrony System (KI D 494/17 and F 477/15).

## Supporting information

Supporting Information

Supporting Information

Supporting Information

Supporting Information
