# Supplementary material for: Respiratory motion prediction based on deep artificial neural networks in CyberKnife system: A comparative study
Source: J Appl Clin Med Phys. 2022 Dec 1;24(3):e13854. doi: 10.1002/acm2.13854 (PMC10018664; doi:10.1002/acm2.13854)
Supplement: Supplementary file 1 — Supporting Information [file ACM2-24-e13854-s002.docx]

**Supplementary Material**

Seven prediction models, including Simple RNN, LSTM, GRU, Bi-simple RNN, Bi-LSTM, Bi-GRU, and CNN-LSTM, are proposed to compensate the manipulator time lag.

1. The RNN network uses a loop to iterate a time-step sequence while maintaining an internal state. In other words, the RNN provides the ability to memorize the internal state to process the sequences of variable length inputs; therefore, the previous outputs are used as inputs. To achieve this goal, first, the previously hidden state information is combined by a vector with the information of the current input. Then, the vector is passed through the activation function, which represents the memory of the network or the new hidden state, to adjust the model's parameter. Finally, the output value is obtained from the rest of the sequence (Figure 1). More information about the RNN structure is described in detail elsewhere [^25^](#_ENREF_25).


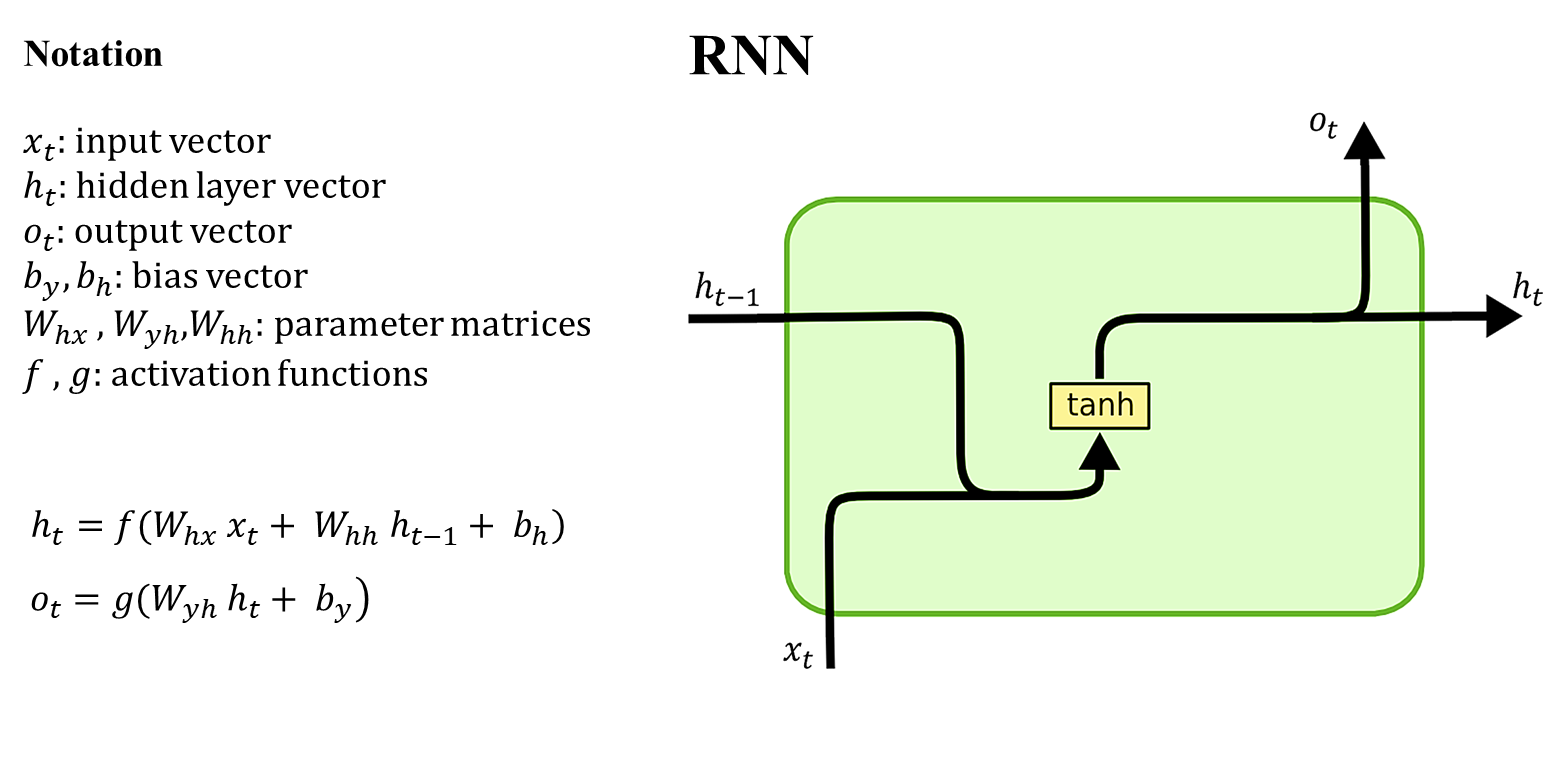


**Figure 1.** The structure of the RNN network. This figure comprises the notation that include input vector, hidden layer, and so on, equation for network structure, and network graph.

1. The LSTM configuration consists of the memory cells and three multiplicative units that decide write, read, and reset operations for the memory cells. These three gates (the input, output, and forget gates) are connected via a nonlinear summation unit. Overall, the proposed LSTM network can be decomposed into four main parts: an input gate, an output gate, a memory cell, and a forget gate (Figure 2). In this context, a detailed explanation of the LSTM model can be found in detail elsewhere [^26^](#_ENREF_26).


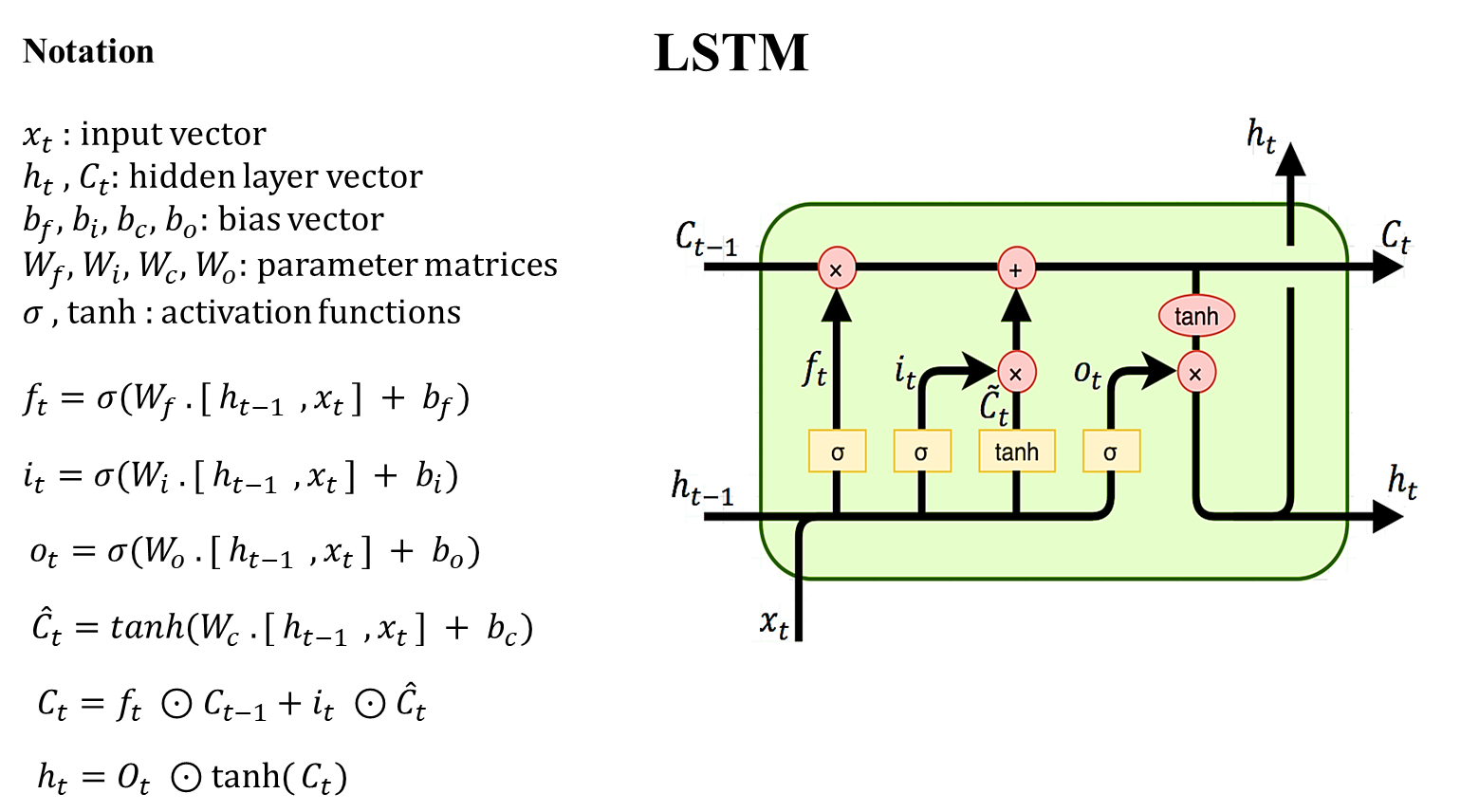


**Figure 2.** The structure of the LSTM network, including a memory cell and three multiplicative units. This figure comprises the notation that include input vector, hidden layer, and so on, equation for network structure, and network graph.

1. The GRU structure, which is similar to the LSTM network, is free of cell state and instead uses a hidden state to transform data. Therefore, the GRU has two gates; an update gate and a reset gate. Whereas the update gate works similarly to the input and forgets gates in the LSTM network, the reset gate decides how much information to forget, discard, or add. In practice, the GRU has few tensor operations; therefore, they are slightly faster during training. For an elaborate description of the GRU network, see Chung et al. [^27^](#_ENREF_27). The GRU structure is shown in Figure 3, including an input gate, a forget gate, and a memory cell.


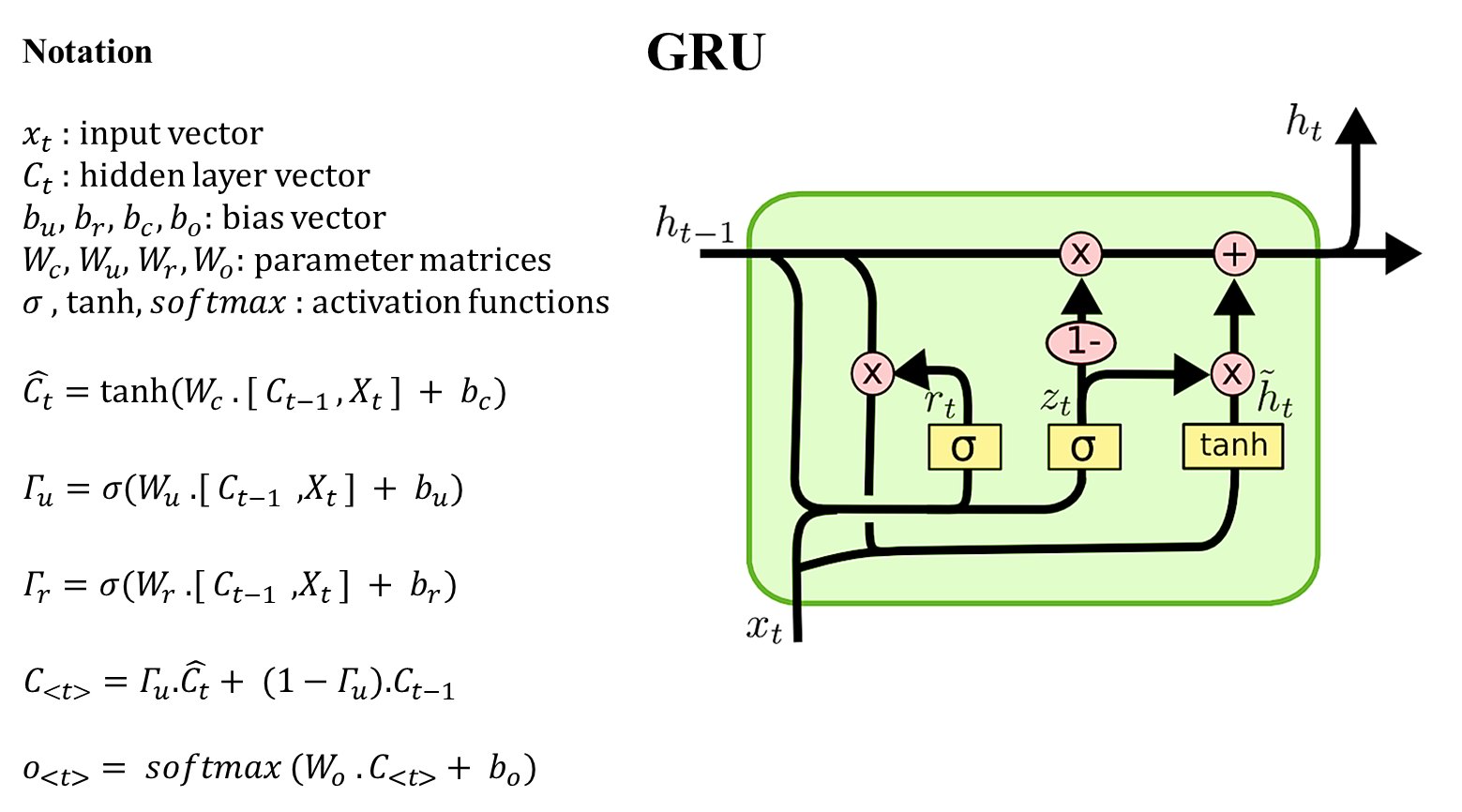


**Figure. 3.** The GRU structure, including a memory cell, an update gate, a rest gate, an updating the hidden layer, and an output gate. This figure comprises the notation that include input vector, hidden layer, and so on, equation for network structure, and network graph.

1. The prediction process in a bidirectional network consists of a forward and backward propagation layer. Whereas the forward propagation layer is used to obtain the prediction value of the historical data, the backward propagation layer is considered to update the internal network parameters. Finally, two layers are connected to a united output layer to get the output value. The detailed information about bidirectional networks is described in detail elsewhere [^28^](#_ENREF_28). In this study, the advantage of a bidirectional network is used to develop three prediction models; Bi-RNN, Bi-LSTM, and Bi-GRU. Also, the basic structure of the bidirectional network is shown in Figure 4.


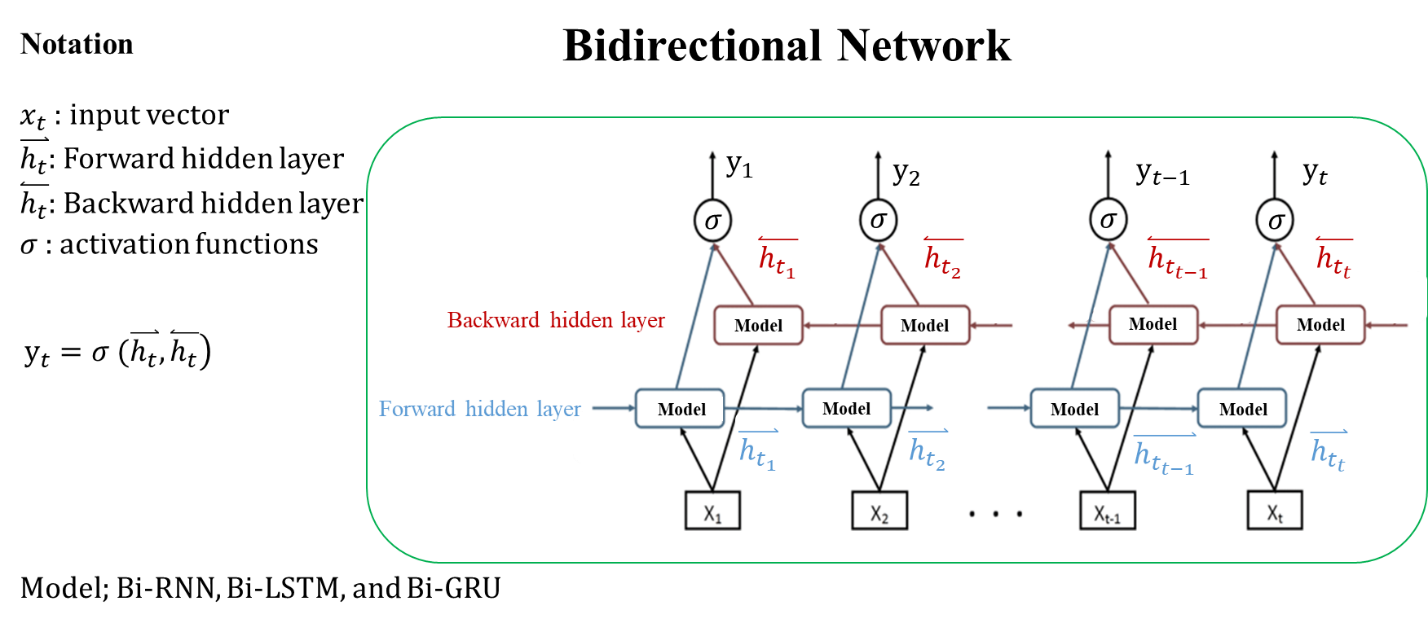


**Figure. 4.** The structure of the Bidirectional Network. This figure comprises the notation that include input vector, hidden layer, and so on, equation for network structure, and network graph.

1. The structure of CNN-LSTM consisted of the CNN network followed by the LSTM model. The CNN model is used for automatic feature extraction and learning from respiration time series data. For this purpose, the input sequences are divided into subsequences, which are processed by the CNN model. The CNN model interprets each subsequence and provides a time series of interpretations of the subsequences to the LSTM model for processing as input. The CNN prediction model used in this study consists of a 1D convolution layer with 30 filters and a kernel size of 1, followed by a MaxPooling1D layer and a Flatten layer that provides data to the LSTM model. The LSTM model consists of one layer with twenty hidden units with a combination of Swish and Tanh activation functions in the hidden and output layers to predict the respiratory signals (Figure 5). More information about the CNN-LSTM structure is described in detail elsewhere [^29^](#_ENREF_29).


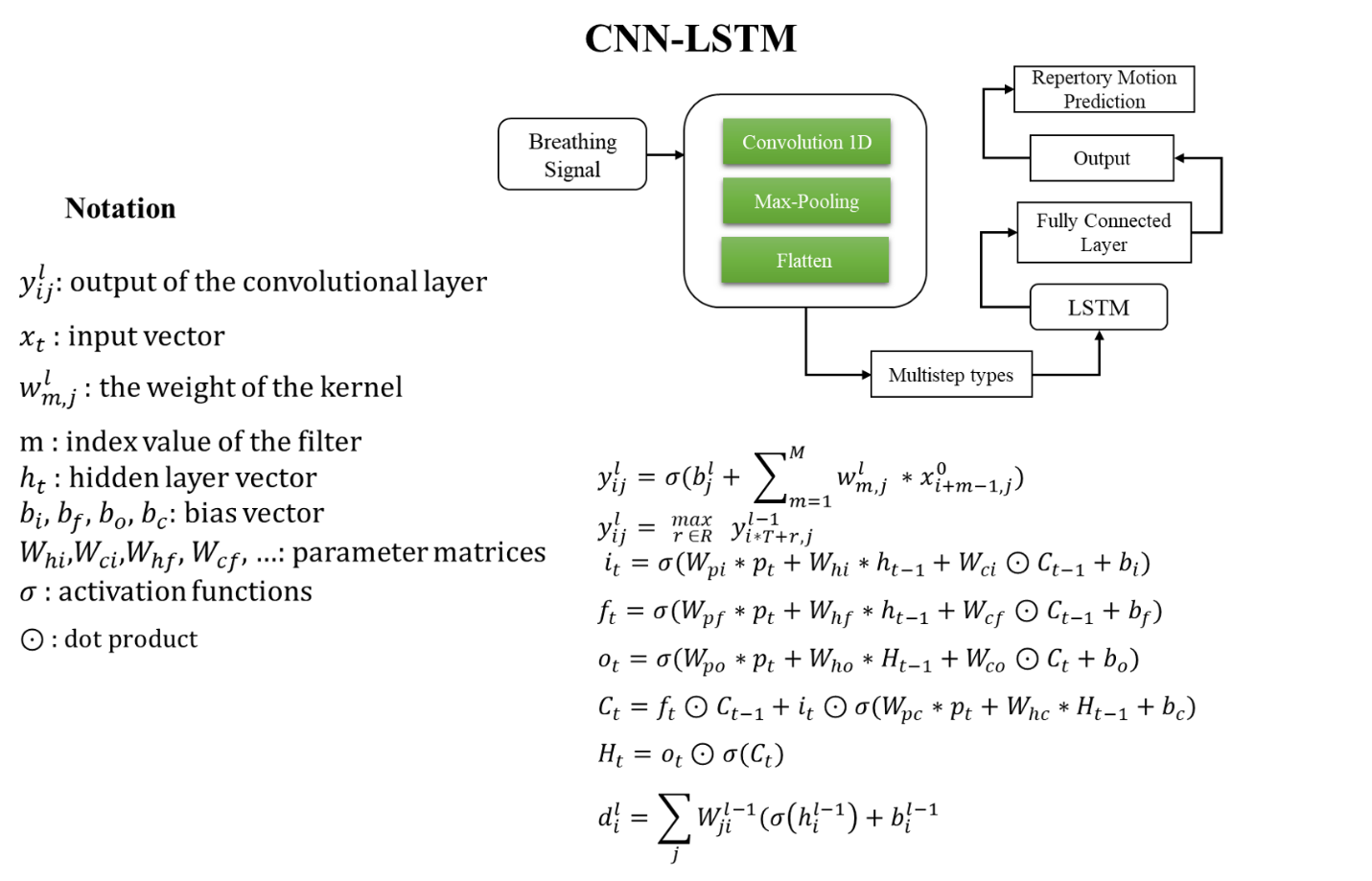


**Figure 5.** The structure of the CNN-LSTM network. This figure comprises the notation that include input vector, hidden layer, and so on, equation for network structure, and network graph.

Twelve variants are used for hyperparameter optimization.

1. Number of layers: The number of layers determines the depth of the model. In this study, the network with 1, 2, 3, and 5 layers was investigated.
2. Number of hidden units in each layer: The number of hidden units represents the number of units per layer selected from the set 3, 5, 10, 20, 30, 40, 50, and 60 units.
3. Optimizer: An optimizer is an important aspect in tuning the network parameters by minimizing the objective function. In this study, seven optimizer methods, including SGD, Adam, Adamax, Nesterov Adam, Adagrad, Adadelta, RMSprop, and Ftrl, were investigated.
4. Learning rate: The learning rate controls how quickly the model is adapted to the problem. Overall, if the learning rate is too low or too high, it results in very slow training or undesirable divergent behavior. In this study, the different ranges of learning rate consist of 0.0001, 0.0005, 0.001, 0.003, 0.005, 0.01, and 0.05 were investigated.
5. The Activation function in hidden and output layers: The activation function is a mathematical gate that determine the output of a network. In this study, different activation functions, including Swish, Sigmoid, Relu, Selu, Elu, Softsign, Tanh, Softmax, Softplus, Hard Sigmoid, And Linear, were considered to investigate the effects of different activation functions in the hidden layers. Also, Linear, Sigmoid, Swish, and Softsign activation functions were considered in the output layer.
6. Number of epochs: The number of epochs is the number of complete passes through the training dataset. This hyperparameter refers to the time taken to process the entire training dataset to run the learning algorithm until the error of the network is suitably minimized. In other words, an epoch is an opportunity to update the internal parameters of the network through a training dataset. Note that a smaller or larger epoch number may result in underfitting or overfitting, respectively. In this study, the range of 125, 250, 500, 1000, and 2000 epoch numbers was investigated.
7. System latency: System latency refers to the latency for data acquisition, saving, reading, beam field adjustment, and robot arm movement. Therefore, the different lengths of system latency may lead to different results. In this study, different ranges of system latency, including 40, 200, 400, and 600 ms, were investigated. Also, five samples represent a system latency of 200 ms.
8. Batch size: Batch size refers to the length of the dataset used before updating the internal model parameters. In other words, the batch size is the number of samples used to predict the value, while the predicted value at the end of the batch is compared with the expected output to calculate the error. Based on this error, the internal model parameters are improved. In this study, the range of 50, 100, 150, 200, 250, 300, 400, and 500 batch sizes was investigated.
9. Loss function: The loss function is used to evaluate the performance of the model by comparing the predicted value with the ground truth value. In this study, four loss functions, including MAE, MSE, Huber, and LogCosh, were considered to evaluate the model’s performance.
10. Input-Slide windowing: The number of data used in the prediction process is called input windowing. In this relation, the set of 1, 5, 10, 20, 30, 50, and 100 windows was used to study the effect of input windowing.
11. Output-Slide windowing: The number of data predicted by the model is called output windowing. In other words, the output slide window is the number of time steps predicted in each step. In this study, the set of 1, 3, 5, and 10 windows was investigated.
12. Multistep types: Mainly two models can be used to forecast the multi-step time series prediction: Vector and encoder-decoder models. Whereas the vector model has been used for direct prediction, the encoder-decoder model provides a type of sequence-to-sequence prediction. In this study, both vector and encoder-decoder approaches are used for both one-step and multi-step prediction.
