# Supplementary material for: Respiratory motion prediction based on deep artificial neural networks in CyberKnife system: A comparative study
Source: J Appl Clin Med Phys. 2022 Dec 1;24(3):e13854. doi: 10.1002/acm2.13854 (PMC10018664; doi:10.1002/acm2.13854)
Supplement: Supplementary file 2 — Supporting Information [file ACM2-24-e13854-s003.pdf]

12 variants are used for hyperparameter optimization.

i. Number of layers: The number of layers determines the depth of the model. In this study, the network with 1, 2, 3, and 5 layers was investigated.

ii. Number of hidden units in each layer: The number of hidden units represents the number of units per layer selected from the set 3, 5, 10, 20, 30, 40, 50, and 60 units.

iii. Optimizer: An optimizer is an important aspect in tuning the network parameters by minimizing the objective function. In this study, seven optimizer methods, including SGD, Adam, Adamax, Nesterov Adam, Adagrad, Adadelta, RMSprop, and Ftrl, were investigated.

iv. Learning rate: The learning rate controls how quickly the model is adapted to the problem. Overall, if the learning rate is too low or too high, it results in very slow training or undesirable divergent behavior. In this study, the different ranges of learning rate consist of 0.0001, 0.0005, 0.001, 0.003, 0.005, 0.01, and 0.05 were investigated.

v. The Activation function in hidden and output layers: The activation function is a mathematical gate that determine the output of a network. In this study, different activation functions, including Swish, Sigmoid, Relu, Selu, Elu, Softsign, Tanh, Softmax, Softplus, Hard Sigmoid, And Linear, were considered to investigate the effects of different activation functions in the hidden layers. Also, Linear, Sigmoid, Swish, and Softsign activation functions were considered in the output layer.

vi. Number of epochs: The number of epochs is the number of complete passes through the training dataset. This hyperparameter refers to the time taken to process the entire training dataset to run the learning algorithm until the error of the network is suitably minimized. In other words, an epoch is an opportunity to update the internal parameters of the network

through a training dataset. Note that a smaller or larger epoch number may result in underfitting or overfitting, respectively. In this study, the range of 125, 250, 500, 1000, and 2000 epoch numbers was investigated.

vii. System latency: System latency refers to the latency for data acquisition, saving, reading, beam field adjustment, and robot arm movement. Therefore, the different lengths of system latency may lead to different results. In this study, different ranges of system latency, including 40, 200, 400, and 600 ms, were investigated. Also, five samples represent a system latency of 200 ms.

viii. Batch size: Batch size refers to the length of the dataset used before updating the internal model parameters. In other words, the batch size is the number of samples used to predict the value, while the predicted value at the end of the batch is compared with the expected output to calculate the error. Based on this error, the internal model parameters are improved. In this study, the range of 50, 100, 150, 200, 250, 300, 400, and 500 batch sizes was investigated.

ix. Loss function: The loss function is used to evaluate the performance of the model by comparing the predicted value with the ground truth value. In this study, four loss functions, including MAE, MSE, Huber, and LogCosh, were considered to evaluate the model's performance.

x. Input-Slide windowing: The number of data used in the prediction process is called input windowing. In this relation, the set of 1, 5, 10, 20, 30, 50, and 100 windows was used to study the effect of input windowing.

xi. Output-Slide windowing: The number of data predicted by the model is called output windowing. In other words, the output slide window is the number of time steps predicted in each step. In this study, the set of 1, 3, 5, and 10 windows was investigated.

xii. Multistep types: Mainly two models can be used to forecast the multi-step time series prediction: Vector and encoder-decoder models. Whereas the vector model has been used for direct prediction, the encoder-decoder model provides a type of sequence-to-sequence prediction. In this study, both vector and encoder-decoder approaches are used for both one-step and multi-step prediction.
